# The RNA-Binding Protein Musashi1 Regulates a Network of Cell Cycle Genes in Group 4 Medulloblastoma

**DOI:** 10.3390/cells11010056

**Published:** 2021-12-25

**Authors:** Mirella Baroni, Gabriela D. A. Guardia, Xiufen Lei, Adam Kosti, Mei Qiao, Tesha Landry, Karl Mau, Pedro A. F. Galante, Luiz O. F. Penalva

**Affiliations:** 1 Children’s Cancer Research Institute, UT Health San Antonio, San Antonio, TX 78229, USA; mibaroni@alumni.usp.br (M.B.); lei@uthscsa.edu (X.L.); akosti@emory.edu (A.K.); ljqmlx@hotmail.com (M.Q.); landryt@uthscsa.edu (T.L.); karl-mau-98@web.de (K.M.); 2 Centro de Oncologia Molecular, Hospital Sírio-Libanês, Sao Paulo 01308-060, Brazil; gguardia@mochsl.org.br (G.D.A.G.); pgalante@mochsl.org.br (P.A.F.G.); 3 Department of Cell Systems and Anatomy, UT Health San Antonio, San Antonio, TX 78229, USA

**Keywords:** medulloblastoma, Musashi1, cell cycle regulation, RNA-binding protein, luteolin

## Abstract

Medulloblastoma is the most common malignant brain tumor in children. Treatment with surgery, irradiation, and chemotherapy has improved survival in recent years, but patients are frequently left with devastating neurocognitive and other sequelae. Patients in molecular subgroups 3 and 4 still experience a high mortality rate. To identify new pathways contributing to medulloblastoma development and create new routes for therapy, we have been studying oncogenic RNA-binding proteins. We defined Musashi1 (Msi1) as one of the main drivers of medulloblastoma development. The high expression of Msi1 is prevalent in Group 4 and correlates with poor prognosis while its knockdown disrupted cancer-relevant phenotypes. Genomic analyses (RNA-seq and RIP-seq) indicated that cell cycle and division are the main biological categories regulated by Msi1 in Group 4 medulloblastoma. The most prominent Msi1 targets include CDK2, CDK6, CCND1, CDKN2A, and CCNA1. The inhibition of Msi1 with luteolin affected the growth of CHLA-01 and CHLA-01R Group 4 medulloblastoma cells and a synergistic effect was observed when luteolin and the mitosis inhibitor, vincristine, were combined. These findings indicate that a combined therapeutic strategy (Msi1 + cell cycle/division inhibitors) could work as an alternative to treat Group 4 medulloblastoma.

## 1. Introduction

Medulloblastoma (MB) is the most frequent malignant tumor of the central nervous system in childhood. Patients with MB are treated with combined modalities involving maximal tumor resection, in conjunction with cytotoxic chemotherapy, and craniospinal irradiation (>3 years old) [1,2]. Most survivors suffer long-term side effects, including neurological, neuroendocrine, and psychosocial deficits [3,4]. In addition, mortality occurs in one-third of the patients due to recurrence of the tumor [5,6]. After several studies that showed the genetic, demographic, and clinical differences of patients with MB, a molecular classification for MB was established [7,8]. Currently, this classification is used by the World Health Organization (WHO), which divides the MB into four main molecular subgroups: WNT, SHH, Group 3, and Group 4 [9]. A more recent study divided MB into 12 different molecular subtypes [10]. Group 4 is the most frequent among MB, but it is the least biologically understood. Some molecular changes are frequently observed, such as the KDM6A mutation, isochromosome 17q, and amplification of the proto-oncogenes *CDK6*, *OTX2*, and *MYCN* [2]. Phosphoproteomic studies have identified potential drivers of Group 4 tumors. These tumors’ phosphoproteomic profiles are defined by the activation of a receptor tyrosine signaling network (*ERBB4*-*SRC*). Based on these results, a Group 4 medulloblastoma mouse model was created via in utero electroporation to deliver a dominant-negative form of Trp53 (DNp53) and an active form of SRC containing a truncated C-terminal domain (SRC-CA). This model resembles G4 MBs and was susceptible to kinase inhibitors [11].

We have defined that another characteristic of Group 4 MB is the high expression of the RNA-binding protein Musashi1 (Msi1). A survival analysis revealed a significant association between Msi1 immunoreactivity and poor overall survival (OS) and progression-free survival (PFS) [12]. Msi1 is a stem cell marker that is essential for the proper development of the nervous system [13,14,15,16]. Msi1 has been shown to function as an oncogenic factor and is highly expressed in several tumors [16,17,18,19,20,21,22]. Studies on different tumor types have established that a decrease in Msi1 expression affects spheroid growth, cell proliferation, survival, apoptosis and tumor growth [12,16,20,21,22,23,24]. Previous genomic studies from our group and others determined that Msi1 regulates hundreds of targets through post-transcriptional mechanisms, affecting numerous cancer pathways, such as NUMB/Notch, PTEN/mTOR, TGFβ/SMAD3, MYC, cMET, and others [25]. This regulation occurs when Msi1 binds to the 3’UTR of its target mRNAs, which causes changes in mRNA stability and/or translation [22].

In this study, we evaluated Msi1 in Group 4 medulloblastoma cell lines, showing that its knockdown disrupts cancer-relevant phenotypes. Genomic analyses (RNA-seq and RIP-seq) showed that cell cycle and cell division are the main biological processes regulated by Msi1 in Group 4 medulloblastoma. Moreover, these cells were susceptible to the Msi1 inhibitor luteolin, by itself and in combination with vincristine, an agent that disrupts mitosis via interaction with microtubules. These results created the basis for a combined therapeutic strategy (Msi1 + cell cycle/division inhibitors) to treat Group 4 medulloblastoma.

## 2. Materials and Methods

### 2.1. Msi1 Expression Levels and Impact on Survival of Medulloblastoma Patients

Msi1 expression analysis in MB subgroups was performed using the Cavalli dataset, which contains a total of 763 MB samples [10]. This analysis was performed using R local scripts. Kaplan–Meier curves were generated to evaluate the correlation between the Msi1 expression levels and overall survival of MB patients from all groups (*n* = 612), Group 4 (*n* = 264), Group 3 (*n* = 113), SHH (*n* = 172) and WNT (*n* = 63) using their default (scan) cut-off. The differences between Msi1 high- and low-expression groups were assessed by log-rank tests. *p*-values smaller than 0.05 were regarded as significant.

### 2.2. Cell Culture

The Group 4 MB cell lines CHLA-01 and CHLA-01R, which derive from the primary tumor and a metastatic site, respectively, were purchased from the American Type Culture Collection (ATCC^®^ CRL3034^TM^). Cells were cultured in suspension in DMEM:F12 Medium with 20 ng/mL human recombinant EGF, 20 ng/mL human recombinant basic FGF, and B27 Supplement to a final concentration of 2% (*v*/*v*), in a humidified atmosphere containing 5% CO_2_ at 37 °C.

Luteolin and Dimethyl sulfoxide (DMSO) were obtained from Sigma-Aldrich, St. Louis, MO, USA (Cat# L9283; Cat# D8418). Vincristine was obtained from Cayman Chemical Company, Ann Harbor, MI, USA (Cat# 11764).

### 2.3. Cell Transfection and siRNA Knockdown

CHLA-01 and CHLA-01R cells were transiently transfected with small interfering RNA (siRNA) via reverse transfection using Lipofectamine RNAiMAX (Invitrogen, Carlsbad, CA, USA; Cat# 13778150) and then used in different assays. Msi1 siRNA SASI_Hs01_00145278 was obtained from Sigma, St. Louis, MO, USA, Msi1 siRNA (Cat# L-011338-00) and ON-TARGETplus Non-targeting control siRNA (Cat# D-001810-01-05) were obtained from Dharmacon (Lafayette, CO, USA). Msi1 knockdown efficiency was determined with quantitative Real-Time PCR (qRT-PCR) and Western blotting.

### 2.4. RNA Extraction, qRT-PCR Analysis, and RNA-Sequencing

CHLA-01 and CHLA-01R RNA was extracted using TRIzol^TM^ reagent (Thermo Fisher Scientific, Grand Island, NY, USA; Cat# 15596018) and cDNA was synthesized using a High-Capacity Kit (Thermo Fisher Scientific, Grand Island, NY, USA; Cat# 4368814) according to the manufacturer’s protocol. Relative levels of mRNA were determined by real-time quantitative PCR using TaqMan Universal PCR Master Mix (Applied Biosystems, Waltham, MA, USA) or PowerUp SYBR Green Master Mix (Applied Biosystems, Waltham, MA, USA). Reactions were performed on ViiA™ 7 Real-Time PCR System (Applied Biosystems, Waltham, MA, USA). Data were analyzed using the 2^−ΔΔCT^ method with GAPDH as an endogenous control. The probes and primers used in qRT-PCR are listed in Table S1.

Samples of CHLA-01R cells were transfected in triplicate with control siRNA or Msi1 siRNA, as described above. RNA samples were obtained as described above and analyzed by RNA-seq. Samples were processed for RNA-seq according to the manufacturer’s instructions (Illumina, San Diego, CA, USA) and sequenced on a HiSeq-3000 machine by the UTHSCSA Genomic Facility.

### 2.5. Western Blotting

CHLA-01R cell pellets were re-suspended and sonicated in the Laemmli sample buffer. Extracted proteins were separated on SDS-PAGE gel and transferred to PVDF membranes. Membranes were blocked in TBS-T + 5% milk and then probed with the following antibodies: Msi1 (Abcam, ab52865), RRM2 (GeneTex, Irvine, CA, USA GTX103193), CDK2 (Cell Signaling, Danvers, MA, USA 2546S), E2F8 (GeneTex, Irvine, CA, USA GTX 112299), CDK6 (Cell Signaling, #3136), RAD51 (Invitrogen, #14419), p27 (Cell Signaling, Danvers, MA, USA #3686), β-tubulin (Sigma-Aldrich, St. Louis, MO, USA), KIAA0101/PCLAF (Abcam ab56773), pSrc (Cell Signaling, Danvers, MA, USA #2101) and Src (Cell Signaling, Danvers, MA, USA #2109). HRP-conjugated goat anti-rabbit antibody (Santa Cruz Biotechnology, Santa Cruz, CA, USA) or HRP-conjugated goat anti-mouse antibody (ThermoFisher, Grand Island, NY, USA) were used as secondary antibodies. Proteins were detected using Immobilon Western Chemiluminescent HRP Substrate (Millipore, Burlington, MA, USA). ImageJ (http://rsb.info.nih.gov/ij/index.html (accessed on 21 September 2021)) was used to compare the densities of bands and to quantify β-tubulin as an endogenous control.

### 2.6. RNA Immunoprecipitation-Sequencing (RIP-Seq)

RIP-Seq experiments were conducted as before [26]. CHLA-01R cell pellets were washed in cold PBS, frozen in dry ice, and stored at −80 °C. To prepare cell lysates, cell pellets were resuspended in 2 volumes of polysomal lysis buffer (KCl 100 mM, EDTA 25 mM, MgCl_2_ 5 mM, HEPES pH7.0 10 mM, NP-40 0.5% and glycerol 10%). Lysates were incubated on ice for 30 min and then sonicated 4× 20 s at 20% amplitude with a 2 min interval. After centrifugation at 15,000 RPM, the supernatant was collected and used later in RIP experiments.

In total, 200 μL of packed Protein A beads (GE Healthcare Life Sciences, Piscataway, NJ, USA) were blocked in NT2 buffer (Tris PH 7.4 50 mM, NaCl 150 mM, MgCl_2_ 1 mM and NP-40 0.05%) + 5% BSA for 30 min at 4 °C, washed and then coated with IgG or anti-Msi1 antibody (Abcam, Cambridge, United Kingdom ab52865). Finally, beads were washed three times in NT2 and then combined with cell extract (5.5 mg of total protein) diluted in 5 volumes of NT2 buffer containing 25 mM EDTA, DTT, VRC, and RNase inhibitor. The solution was rotated at room temperature for 3 h and then centrifugated at 2000 RPM in a tabletop centrifuge for 5 min at 4 °C. The supernatant was discarded, and beads were washed five times with 1 mL of NT2 buffer. RNA was phenol extracted and then purified using the RNasey MinElute Cleanup kit (Qiagen, Hilden, Germany) following the manufacturer’s instructions. Eluted RNA samples were analyzed by RNA-seq.

### 2.7. RNA-Sequencing and RIP-Seq Analyses

Using FASTq files from RNA-seq assays, transcript quantification was performed using Kallisto (v0.43.1, parameters: single −l 51–s 1 × 10^−8^ [27], with the insert metrics obtained from the library construction. Gene-level counts were obtained by using Kallisto transcript quantification as input to the R package tximport (v1.0.3) [28]. GENCODE (v29, gencodegenes.org) was used as reference for the human transcriptome. Differential gene expression analysis was performed with DESeq2 v3.6.2 [29]. Genes were classified as differentially expressed using an adjusted *p*-value < 0.05 and |log2FoldChange| ≥ 0.5.

Samples of CHLA-01R cells were immuno-precipitated with either IgG-coated beads (control) or anti-Msi1-coated beads. The samples were prepared in triplicate and analyzed by RIP-seq. To identify transcripts preferentially associated with Msi1, we compared experimental samples to controls, and used log2FoldChange ≥ 0.5 and an FDR-adjusted *p*-value < 0.05 as the cut-offs. Raw datasets were submitted to the EBI-ENA resource, accession number PRJEB40550.

### 2.8. Gene Ontology and Network Analyses

Gene Ontology (G.O.) enrichment analysis was performed by using the webtool PANTHER statistical overrepresentation test webtool (pantherdb.org (accessed on 21 September 2021)) [30]. For all analyses, the whole human genome was used as background. For a summarized G.O. term selection, we used REVIGO (revigo.irb.hr (accessed on 21 September 2021)) [31]. KEGG’s pathways enrichment analysis was performed using the ShinyGO webtool (bioinformatics.sdstate.edu/go/ (accessed on 21 September 2021)) [32]. In both analyses, terms and pathways with false-discovery rate values smaller than 0.05 were considered enriched.

HumanBase [33] was employed to identify correlated gene modules in the RNA-seq and RIP-seq datasets. HumanBase applies community detection to obtain cohesive gene clusters. It is based on shared k-nearest-neighbors (SKNN) and the Louvain community-finding algorithm to cluster modules of tightly connected genes. The central nervous system was selected as the tissue to conduct the analyses.

We used the STRING database [34] to construct protein–protein interaction networks and determine associations among cell cycle genes identified in the RNA-seq and RIP-Seq analyses. The interactions are based on experimental evidence procured from high-throughput experiments, text mining, and co-occurrence.

### 2.9. IncuCyte Analysis

CHLA-01 and CHLA-01R grow as cell aggregates that resemble spheroids. To grow cells as monolayers, 40µL of Geltrex^TM^ basement membrane matrix (Thermo Fisher Scientific; Cat# 12760013) was used to coat plates. CHLA-01R cells were plated onto 96-well plates (3000 cells/well) coated with Geltrex^TM^ and then treated with DMSO (control) or different concentrations of luteolin. Plates were transferred to the IncuCyte automated microscope system (Essen BioScience, Ann Harbor, MI, USA) and cells were counted (four images per well) every 2 h for 7 days. All experiments were performed in triplicate.

### 2.10. Cell Growth

Cells were transfected with siRNAs and plated onto 96-well plates (5000 cells/well). Plates were transferred to the IncuCyte automated microscope system (Essen BioScience, Ann Harbor, MI, USA) and the number of colonies was counted after 3 days. All experiments were performed in triplicate.

### 2.11. MTS Assay

CHLA-01R cells were transiently transfected with siRNAs and plated onto 96-well plates (3000 cells/well) coated with Geltrex^TM^ and incubated at 37 °C for 72 h. Next, 20 μL of MTS mixture (1000 μL MTS and 50 μL PMS) was added to each well and samples were incubated at 37 °C for an hour. Optical density was measured at an absorbance of 490 nm with a Synergy HT microplate reader (BioTek, Winooski, VT, USA). MTS was also added to the cells treated with luteolin, vincristine, or DMSO for 72 h, and absorbance was measured. All experiments were performed in triplicate.

### 2.12. Caspase-3/7 Assay

Transfected CHLA-01R cells were plated into 96-well opaque plates, and after 72 h, 100 µL of Caspase-Glo^®^ 3/7 Reagent (Caspase-Glo^®^ 3/7 Assay System-Promega, Madison, WI, USA) was added to each well and incubated at room temperature for 1 h. Luminescence was measured for each well by a plate-reading luminometer. The same procedure was performed with cells treated with DMSO or luteolin. All experiments were performed in triplicate.

### 2.13. Cell Cycle Assay

Cell cycle analysis was performed by flow cytometry after Propidium Iodide (PI) staining. siControl and siMsi1 CHLA-01 and CHLA-01R cells were plated into six-well plates (6 × 10^4^ cells/well) and, after 72 h, cells were harvested, washed with cold PBS twice, fixed with 70% cold ethanol solution, and then kept at −20 °C overnight. Cell pellets were resuspended in PBS with RNAse A (10 ng/mL) and incubated at 37 °C for 30 min. Next, cells were centrifuged, and PBS with PI was added at a final concentration of 50 μg/mL for staining. After incubation, cell cycle analysis was conducted in a FACS BD caliber. All experiments were performed in triplicate.

### 2.14. Statistical Analysis

Statistical analyses were performed using GraphPad Prism 8.0 software. For gene expression analysis, the Mann–Whitney test was used, and for the analysis of functional assays one-way ANOVA, a two-way ANOVA, or *t*-test were used. A threshold with a *p*-value < 0.05 was defined as statistically significant.

### 2.15. Drug-Drug Interaction

The Combination Index (CI) for drug–drug interactions was calculated to find out whether the combination of the drugs luteolin and vincristine is synergistic or antagonistic. The general equation is CI = AB/(A × B), where: AB = measured values for combined treatment/control (DMSO) and A and B = values for the single treatment and control. Thus, CI < 1 indicates that the combination treatment is synergistic and CI > 1 indicates that the combination is antagonistic [35].

## 3. Results

### 3.1. High Expression of Msi1 Is Associated with a Worse Prognosis in Group 4 MB

**Figure 1:**
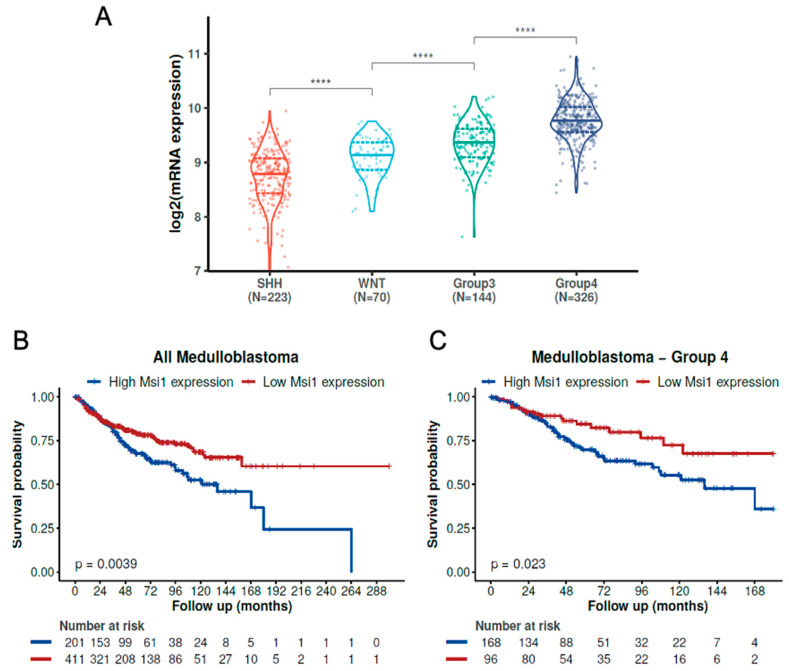
**High Musashi1 expression is associated with a worse prognosis in Group 4 medulloblastoma**. (**A**) Msi1 expression in MB molecular subgroups (**** *p* < 0.0001; Wilcoxon tests). Kaplan–Meyer curves show the impact of Msi1 expression levels on the survival of all MB patients (**B**) and only Group 4 MB patients (**C**) from the Cavalli cohort [10]. All significant comparison (*p*-value < 0.001; Wilcoxon test) are presented by “****”.

We have previously shown that high expression of Msi1 is prevalent in medulloblastoma groups 3 and 4 [12]. To confirm and expand this analysis, we evaluated the Msi1 expression in the Cavalli dataset, which contains 763 samples of MB [10]. The results showed that Msi1 is highly expressed in groups 3 and 4, having the highest expression level in Group 4—Figure 1A. Next, we determined the impact of Msi1 levels on patient survival. High Msi1 expression was associated with a worse prognosis, considering all MB patients (Figure 1B) and only patients of Group 4 (Figure 1C). The impact of a high Msi1 expression was also associated with a worse prognosis in the MB group WNT (Figure S1), but not significant in the MB group SHH and MB Group 3. Regardless, we found the same trend of worse survival in patients with a higher Msi1 expression in the latter two groups (Figure S1).

### 3.2. Msi1 Knockdown Affects Cancer-Relevant Phenotypes

The analysis of Group 4 medulloblastoma is challenging due to the scarce number of cell lines. There are only two available lines, CHLA-01 and CHLA-01R, which are from the same patient and derive from primary tumor and metastasis, respectively.

**Figure 2:**
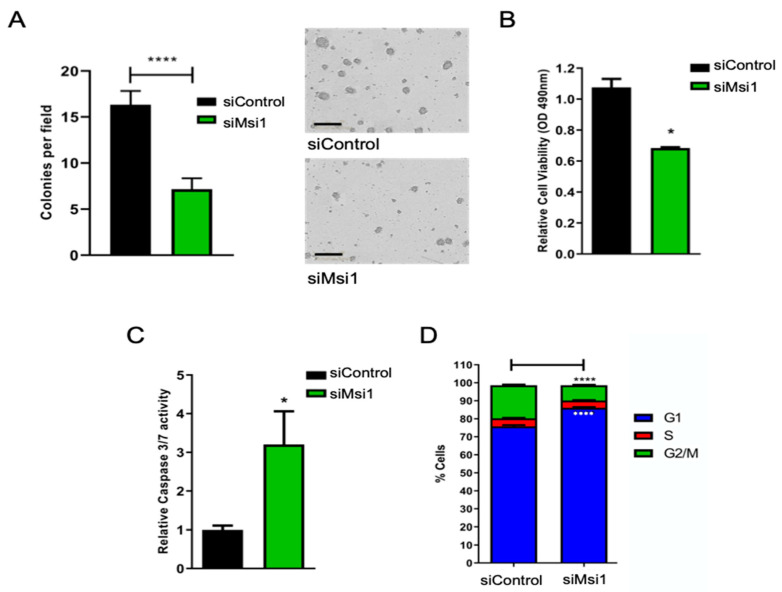
**Musashi1 knockdown affects cancer-related phenotypes in CHLA-01R cells**. (**A**) Effect of Msi1 knockdown on cell growth (A), cell viability according to the MTS assay (**B**), apoptosis verified by caspase assay (**C**) and cell cycle distribution shown in FACS-sorted cells (**D**). Statistical significance calculated by one-way ANOVA and t test. Data shown as means ± S.D. (* *p* < 0.05, **** *p* < 0.0001).

Msi1 knockdown impaired cell growth, as noted by the lower number and reduced size of colonies (Figure 2A and Figure S2A). Similarly, a reduction in Msi1 levels led to a decrease in cell viability, as shown by MTS (Figure 2B), and an increase in caspases-3 and -7 activity, suggesting higher apoptosis levels (Figure 2C). Consistent with our previous observations in glioblastoma [25], Msi1 silencing altered cell cycle progression, leading cells to arrest in the G1 phase (Figure 2D and Figure S2B).

### 3.3. Msi1 Regulates the Expression of Cell Cycle and Division Genes

**Figure 3:**
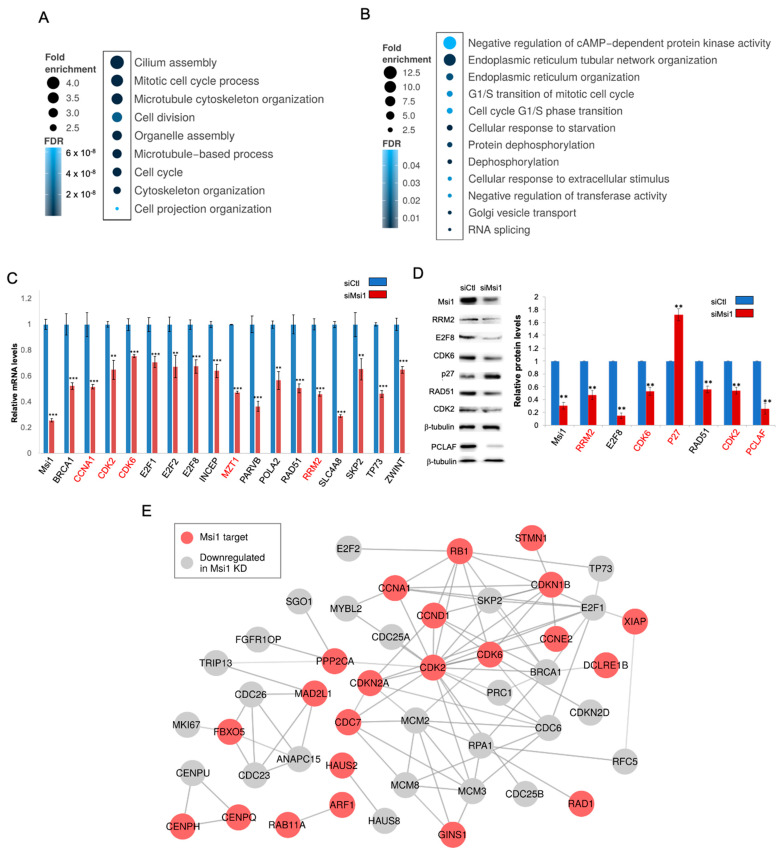
**Musashi1 regulates a network of genes implicated in cell cycle/division in medulloblastoma G4 cells**. Gene ontology-enriched terms (biological processes) associated with genes downregulated after Msi1 knockdown (**A**) and Msi1 targets (**B**) identified by RNA-seq and RIP-seq, respectively. GO analysis was conducted using Panther [30] and terms were compiled with REVIGO [31]. Expression of cell cycle/division genes in Msi1 knockdown cells (siMsi1) vs. control cells (siCtl) by qRT-PCR (**C**) and Western blot (**D**). ImageJ (http://rsb.info.nih.gov/ij/index.html (accessed on 21 September 2021)) was used to compare the densities of bands and to quantify using β-tubulin as an endogenous control. Genes that were also identified as Msi1 targets are labeled in red. Statistical significance was calculated by multiple *t*-test (** *p* < 0.01, *** *p* < 0.001). (**E**) Protein–protein network according to STRING [34] showing cell cycle/division genes identified as Msi1 targets or downregulated in Msi1 knockdown cells. Msi1 targets (in red) were main nodes. The second layer contains genes downregulated in Msi1 knockdown cells.

To identify the main biological processes and pathways regulated by Msi1 in Group 4 MB, we conducted RNA-seq and RIP-seq experiments in CHLA-01R cells. A gene ontology analysis showed that genes downregulated upon Msi1 knockdown are strongly associated with cell cycle/division—Figure 3A and Table S2. Enriched GO terms associated with the cytoskeleton have also been identified—in particular, mechanisms related to the organization of microtubules in mitosis—Figure 3A and Table S2. A disturbance in chromosome segregation can cause genomic instability, so the attachment between microtubules and chromosome centromeres is essential for the correct occurrence of cell division. Interestingly, we found in GBM that Msi1 regulates the expression of members of the centromeric complex, which resulted in an increase in mitotic catastrophe and changes in cell cycle distribution [36].

The RIP-seq analysis identified 936 coding transcripts preferentially associated with the Msi1 protein, and 216 of them were also identified in other cell lines in CLIP- and RIP-seq studies performed by our lab [12,25,37]—Table S3. Similar to what was observed in the analysis of genes downregulated after Msi1 knockdown, we identified several transcripts implicated in cell cycle regulation—Figure 3B and Table S3. Many cell cycle/division genes that showed expression alteration in Msi1 knockdown CHLA-01R cells were not identified as Msi1 targets in this or other studies [12,25,37] and are likely the result of indirect regulation. The E2F family of transcription factors is known to be critical in cell cycle regulation (62), and thus may be driving the expression of this set of genes. Msi1 knockdown caused a decrease in the expression of E2F1, E2F2 and E2F8 (Table S2, Figure 3C). Previously, we observed a downregulation of E2Fs in Msi1 KO glioblastoma cells and established them as the main drivers of Msi1 impact on cell cycle/division genes [36]. In addition, transgenic mice with Msi1 overexpression in the intestine showed an increased expression of these transcription factors [38]. E2Fs are still poorly characterized in the context of medulloblastoma but their role in cell cycle has been well established in many other tumor types [39]. According to the Cavalli dataset [10], the high expression of E2F1, E2F2 and E2F8 correlates with poor prognostic outcomes in medulloblastoma patients. CDKN2A (p16) and CDKN1B (p27), which are negative regulators of E2F transcription factors [36], were determined to be targets of Msi1 in our study via RIP-Seq (Table S3). In fact, it has been previously shown that Msi1 represses the translation of p16 and p27 [23,40,41]. We suggest then that by repressing p16 and p27, Msi1 would lead to an increase in E2F1, E2F2, and E2F8 expression and the subsequent activation of cell cycle genes.

The impact of Msi1 on the expression of cell cycle/division genes was corroborated by qRT-PCR and Western blotting—Figure 3C,D and Figure S3A. Other biological processes enriched among identified Msi1 targets include splicing, the regulation of kinase activity, endoplasmic reticulum organization, and protein dephosphorylation—Figure 3B and Table S3. When comparing RIP-seq and RNA-seq results, we observed that Msi1 targets appeared more often in the downregulated set (Figure S3B–E), suggesting that the regulation of mRNA stability might be the preferential mechanism of action for Msi1.

We performed a second analysis with HumanBase [33] to expand on biological processes and pathways preferentially regulated by Msi1 in Group 4 medulloblastoma. We identified three gene modules associated with downregulated genes upon Msi1 knockdown and seven modules associated with Msi1 targets identified by RIP-Seq—Table S4. We then conducted Gene Ontology (GO) analyses of these gene modules and, after comparison, we identified several shared enriched GO terms including cell cycle and division, DNA replication, and repair—Table S4.

**Figure 4:**
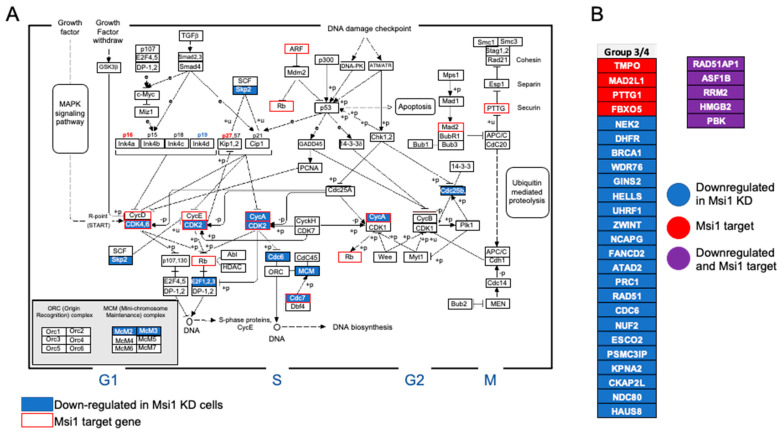
**The impact of Msi1 on the cell cycle shown on the KEGG’s cell cycle pathway**. (**A**) Diagram of the cell cycle pathway illustrating the impact of Msi1 regulation. Msi1 targets identified by RIP-seq are labeled in red while downregulated genes after Msi1 knockdown, identified by RNA-seq, are labeled in blue. (**B**) Targets of Msi1 and/or genes downregulated in Msi1 knockdown cells featured as biomarkers of a cell population present in subtypes 3/4-α of MB, characterized by the amplification of cell cycle activity [42].

We built a cell cycle/division network with Msi1 target genes identified in the RIP-seq analysis and genes downregulated in Msi1 knockdown cells; CDK2, CDK6, and Cyclin D1 were identified as main nodes (Figure 3E). In Figure 4A, we put the results into perspective by mapping Msi1-regulated genes to the KEGG’s cell cycle pathway. The large number of genes connected to G1 supports results showing that Msi1 knockdown in CHLA-01R and CHLA-01 cells led to G1 arrest. In support of Msi1’s role as a regulator of cell cycle and division in Group 4 MB, we saw, in a single-cell study evaluating intertumoral heterogeneity in MB subgroups, a signature associated with cell populations in subgroups 3–4a [42]. This subset of cells is strongly associated with the cell cycle and contains multiple Msi1 targets and genes downregulated upon Msi1 knockdown in medulloblastoma cells (Figure 4B and Table S5).

### 3.4. A Link between Msi1 and SRC Signaling

Aberrant ERBB4-SRC signaling was defined as a driver of Group 4 MB (11). ERBB4 is a tyrosine kinase receptor and is critical during cerebellum and medulloblastoma development (43), while SRC signaling controls important biological/oncogenic processes, including proliferation, apoptosis, cell adhesion and motility (44). We have previously identified SRC as an Msi1 target [25], but it was not detected in the RIP-seq analysis performed on CHLA-01R cells. However, a network analysis identified several Msi1-regulated genes associated with SRC, including *CCNA1*, *NOX1*, *EFNA5*, *IGF2BP1*, *LRP6*, *RND2*, *CDKN2A*, *NGF*, *EFNA5*, and *EFNB3*—Figure S4A. From this list, *CCNA1*, *NOX1*, *CDKN2A*, and *RND2* show higher expression in Group 4, like Msi1. Moreover, a gene expression correlation analysis using the Cavalli dataset [10] indicated that Msi1 displays a high correlation with both SRC and ERBB4 in medulloblastoma samples—Figure S4B. Finally, we determined that Msi1 knockdown in CHLA-01R cells causes a decrease in SRC and p-SRC levels—Figure S4C. The changes in p-SRC levels were even more pronounced, suggesting that Msi1 could also influence SRC phosphorylation. In fact, among the identified Msi1 targets are several proteins known to influence SRC phosphorylation, including PTP4A1, KRAS, PPP2CA, and ITGB1.

### 3.5. Msi1 Knockdown Promotes the Expression of Genes Implicated in Morphogenesis and Development

We found 199 upregulated genes after knocking down Msi1—Figure S5 and Table S2. We conducted a gene ontology analysis and compared results to the ones obtained in a study with GBM Msi1 knockout lines [36]. Although the number of genes determined to be upregulated in both studies was relatively small, we ended up identifying several common GO terms related to development, morphogenesis, and cell adhesion—Figure S5 and Table S2.

MB originates from neural progenitors and the activation of developmental pathways in these cells is critical for normal cerebellar morphogenesis [43]. Our results indicate that high Msi1 expression represses these pathways. We should highlight genes that were found upregulated in both of our MB and GBM studies: BBC3, EGLN1, IGFBP5, MSX1, NDRG2, and PHLDA3. These genes, when overexpressed, are associated with tumor inhibition and/or better prognosis in different tumors [44,45,46,47,48,49,50,51,52,53,54]. Therefore, their “repression” by Msi1 could be an important component of Msi1 contribution to MB Group 4 development. One example is the homeobox gene, MSX1 [55]. High MSX1 activity is associated with the inhibition of migration and proliferation in different tumor types [49,50]. Another important tumor suppressor gene showing increased expression after Msi1 silencing is NDRG2, an n-MYC target gene, which is highly expressed in normal tissues, but undetectable in many tumors [51]. Groups 3 and 4 are characterized in part by NMYC and MYC-driven MBs [56]. Therefore, NDRG2 downregulation may be a crucial step in the context of these subgroups. In GBM, NDRG2 is frequently inactivated and influences patient survival [52,53]. Another relevant gene identified in our analysis is PHLDA3, which was defined as a potent inhibitor of the Akt pathway [54,57]. In Group 4 MB, high Akt pathway activity has been shown to impact patient prognosis [58].

### 3.6. Luteolin (Msi1 Inhibitor) Inhibits Proliferation and Sensitizes MB Cells to Vincristine Treatment

**Figure 5:**
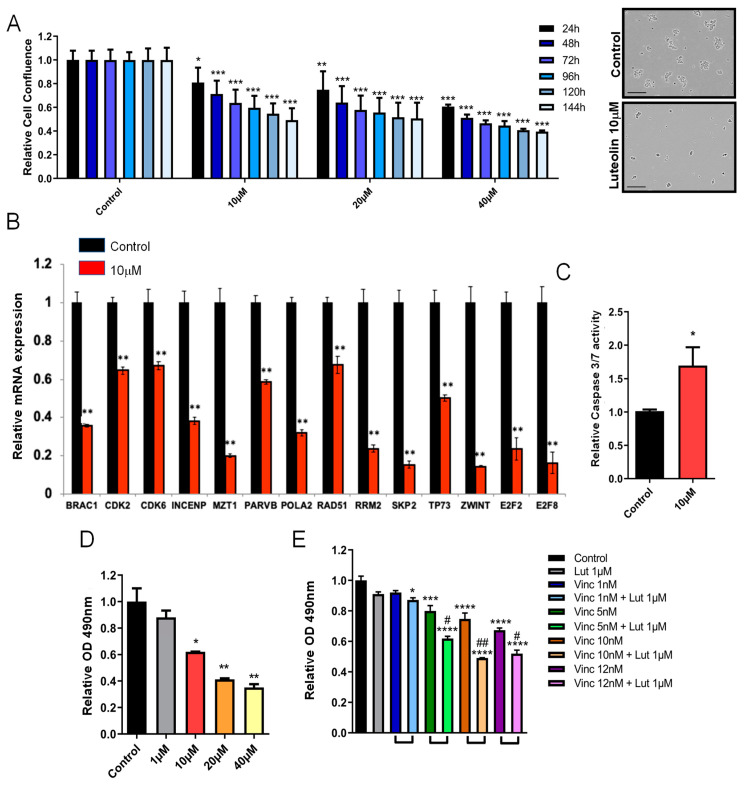
**Musashi1 inhibitor, luteolin, impairs the growth of CHLA-01R cells**. (**A**) Cell number over time of CHLA-01R cells treated with luteolin according to the automated Incucyte system and picture showing the aspect of control vs. luteolin-treated cells (**B**) Expression of cell cycle/division genes by qRT-PCR and (**C**) apoptosis analysis using the caspase assay after luteolin treatment (10 µM). (**D**) Cell viability measured with the MTS assay. (**E**) Results of the MTS of cells treated with luteolin, vincristine or combination. The Combination Index (CI) for drug–drug interaction [35] was calculated and it was determined that luteolin and vincristine work synergistically. Statistical significance was calculated by one-way ANOVA and *t*-test. Data shown as means ± S.D. (* *p* < 0.05, ** *p* < 0.01, *** *p* < 0.001 **** *p* < 0.0001, ^#^ CI < 0.9, ^##^ CI < 0.8).

We previously described luteolin as an inhibitor of Msi1 and showed that it blocks Msi1 regulatory functions in glioblastoma cells and impairs their growth [59]. We evaluated whether luteolin could be used as an agent to treat Group 4 medulloblastoma. CHLA-01R cells treated with luteolin showed a reduction in cell number over time, in the expression of cell cycle genes, as shown by qRT-PCR, and in cell viability, as detected by MTS. On the other hand, luteolin-treated cells showed increased apoptosis as indicated by Caspase 3 assay—Figure 5A–D. A reduction in cell number over time was also observed in CHLA-01 cells treated with luteolin—Figure S2C.

Monotherapies are unlikely to be effective in cases of aggressive tumors such as Group 4 MB. We have previously shown that luteolin functions synergistically with radiation and the PARP inhibitor olaparib in glioblastoma cells [59]. Considering the impact of Msi1 on cell cycle/division, we chose to combine luteolin and vincristine, a chemotherapy medication used to treat MB that disrupts mitosis via the interaction with microtubules. Vincristine is highly toxic and, when used in combination with drugs, could help lower the dosage, benefiting patients. We observed that a low dose of luteonin was able to enhance the effect of vincristine on cell viability after 72 h of treatment. The Combination Index (CI) [35] for drug–drug interactions was calculated and the results indicated that the drugs functioned synergistically—Figure 5E.

## 4. Discussion

Musashi1 belongs to a highly conserved family of stem cell proteins with orthologues described in many invertebrate and vertebrate species [60,61,62,63,64]. In humans, two very similar proteins have been described, Musashi1 and Musashi2, which display almost identical RNA-binding domains [60,61,62,64]. In the nervous system, Musashi proteins regulate the balance between self-renewal and differentiation and are required for proper brain development [60,61,62]. Although they seem to have redundant functions in the nervous system, the results of the analysis of Msi1 KO mice indicated that Msi2 cannot fully replace Msi1 [65]. In the cancer scenario, both proteins behave as oncogenic factors and have been characterized in many tumor types [16,20,21,22]. However, there are differences in relation to their expression patterns and roles [21]. For instance, both Msi1 and Msi2 have been implicated in colon cancer, but Msi1 and Msi2 have unique ties to glioblastoma and leukemia, respectively [21,22]. In the particular case of medulloblastoma, Msi1 and Msi2 show very distinctive expression patterns in MB subgroups and high Msi2 expression does not show any impact on disease prognosis. Msi1 expression is particularly high in Group 4, and we showed here that it correlates with poor prognosis in this particular group of patients. Previous analyses of Msi1 in MB were performed only in SHH cells [12,23,24] and, therefore, a study specifically on Group 4 as presented here was in demand.

Msi1 has been shown to bind to a short motif with an invariable UAG at the center that tends to be localized in stem-loop structures [66]. Although present in different regions, Msi1 binding sites are preferentially located at 3’ UTRs, agreeing with its described roles in translation regulation and mRNA stability. Additional Msi1 functions have been recently described that include its participation in stress granules and the formation of tau aggregates [67,68]. Targets of Musashi proteins have been identified in many different cell types by CLIP and RIP and encompass a variety of cancer-related processes and pathways [12,25,37,69]. Circa 25% of the Msi1-associated transcripts we identified in CHLA-01R cells were also found in other cell lines [12,25,37]. This group of “core Msi1 targets” is associated preferentially with processes such as endoplasmic reticulum and vesicle organization, response to stress and protein modification and localization.

### 4.1. Musashi1 as a Main Driver of the Cell Cycle and Division in Group 4 MB

Group 4 is the most common MB subgroup and its expression signature shows enrichment for neuronal development pathways [10], in which Msi1 is known to play fundamental roles [56,70]. The identification of oncogenic drivers of Group 4 MB has been challenging. In a single-cell transcriptomics study, Group 4 tumors were found to be very heterogeneous; three main distinct transcriptional programs were identified: Group 3/4-A, -B, and -C [42]. Group 3/4-A is characterized by markers of cell cycle activity [42]. Msi1 could have a role in controlling this transcriptional program as many genes in this signature are regulated by Msi1 according to our genomic analyses.

Cell cycle/division was the common denominator between the RIP-seq and RNA-seq analyses, suggesting that these processes are the main routes used by Msi1 to contribute to Group 4 medulloblastoma. In a previous study where we generated a transgenic mouse line overexpressing Msi1 in the intestine, we showed that Msi1 ectopic expression increased the levels of a large number of cell cycle genes [38]. Similarly, we showed that the regulation of cell cycle and division is the main route for Msi1 of contribution to glioblastoma development [36]. Although Msi1 targets are connected to different phases of the cell cycle, the majority are associated with the G1-S transition. In agreement with this, we observed that Msi1 knockdown produced G1 arrest and the same effect was observed in other studies [25,41,71,72]. Among the G1-related Msi1 targets that were identified in different studies, we should highlight CCND1, RB1, CDK6, and CDKN2A.

### 4.2. Oncogenic RBPs as Possible Therapeutic Targets

RBPs can regulate several cellular processes and alterations in RBP expression can lead to various diseases, including cancer. Many oncogenic RBPs such as hnRNPH1, IGF2BP3, HuR, PTB, and SNRPB have been identified and characterized in the context of brain tumors [73,74]. Due to their broad impact on gene expression and the possibility of identifying inhibitors tailored to their unique RNA-binding domains, RBP targeting has started to be explored in cancer therapy [75].

Luteolin was the top hit in a high-throughput screening to identify compounds capable of blocking the RNA-binding properties and functions of Msi1. Subsequently, we showed the anti-tumorigenic effect of luteolin in GBM cells and its possible use in combination with radiation and the PARP inhibitor olaparib [59]. Other studies showed the chemo-sensitizing effect of luteolin in different tumors such as ovarian cancer [76,77,78], gastric cancer [79] and hepatocellular carcinoma [80]. The combination of luteolin with other agents, for instance, with cisplatin in the case of ovarian cancer, showed to be an effective strategy to prevent tumor growth [78]. We successfully tested the potential use of luteolin in Group 4 as a therapeutic agent. Vincristine, a compound that binds microtubules, impairing cell division [81], is often used in medulloblastoma treatment but commonly produces undesirable side effects [2]. The combined use of other drugs interacting with vincristine would be an option to maintain treatment efficiency while decreasing side effects. With Msi1’s impact on the expression of cell division genes in mind, we tested luteolin + vincristine combinations and observed a synergistic interaction that could be explored in therapy.

## 5. Conclusions

We established that high expression of Msi1 is often observed in medulloblastoma Group 4 patients and is linked to poor survival. Genomic analyses followed by biological assays suggested that the main contribution of Msi1 in these tumors is to regulate a network of cell cycle/division genes. Finally, we determined based on in vitro results that combination therapy (vincristine + luteolin/Msi1 inhibitor) might be a viable alternative to treat Group 4 medulloblastoma patients.

## Data Availability

Data supporting the findings of this study are available within the article and in S. Raw datasets were submitted to the EBI-ENA resource, accession number PRJEB40550.

## Supplementary Materials

The following supporting information can be downloaded at: https://www.mdpi.com/article/10.3390/cells11010056/s1, Figure S1: Kaplan–Meyer curves showing the impact of Msi1 expression levels on the survival of MB patients from Groups WNT, SHH and 3, Figure S2: The impact of Msi1 on CHLA-01 cells phenotypes, Figure S3: Confirmation and validation of Msi1 knockdown in CHLA-01 and CHLA-01R cells and combined analysis of RNAseq and RIPSeq results, Figure S4: The relationship of Musashi1 with SRC and ERBB4, Figure S5: Musashi1 regulates a network of genes implicated in development/morphogenesis in medulloblastoma G4 cells, Table S1: List of primers and probes used in qRT-PCR analysis, Table S2: Genes altered in CHLA-01R Msi1 knockdown cells identified by RNA-seq, Table S3: Musahi1 target genes in CHLA-01R cells identified by RIP-seq, Table S4: Gene modules identified with HumanBase [33], Table S5: List of genes that were characterized as biomarkers of different subtypes of MB according to Hovestadt et al., 2019 [42].
