# Supplementary material for: The RNA-Binding Protein Musashi1 Regulates a Network of Cell Cycle Genes in Group 4 Medulloblastoma
Source: Cells. 2021 Dec 25;11(1):56. doi: 10.3390/cells11010056 (PMC8750343; doi:10.3390/cells11010056)
Supplement: Supplementary file 1 [file cells-11-00056-s001.zip › cells-1410433 supple/Sup Material/Sup material.pdf]

cells

MDPI

Article

The RNA binding protein Musashi1 regulates a network of cell cycle genes in Group 4 medulloblastoma

Mirella Baroni[1], Gabriela A. D. Guardia[2], Xiufen Lei[1], Adam Kosti[1,3], Mei Qiao[1], Tesha Landry[1], Karl Mau[1], Pedro A. F. Galante[2], Luiz O. F. Penalva[1,3*]

1 Children's Cancer Research Institute, UT Health San Antonio, San Antonio, Texas, 78229, USA
2 Centro de Oncologia Molecular, Hospital Sírio-Libanês, São Paulo, São Paulo, 01308-060, Brazil
3 Department of Cell Systems and Anatomy, UT Health San Antonio, San Antonio, Texas, 78229, USA
* Correspondence: penalva@uthscsa.edu;

SUPLEMENTARY MATERIAL

SUPPLEMENTARY FIGURES

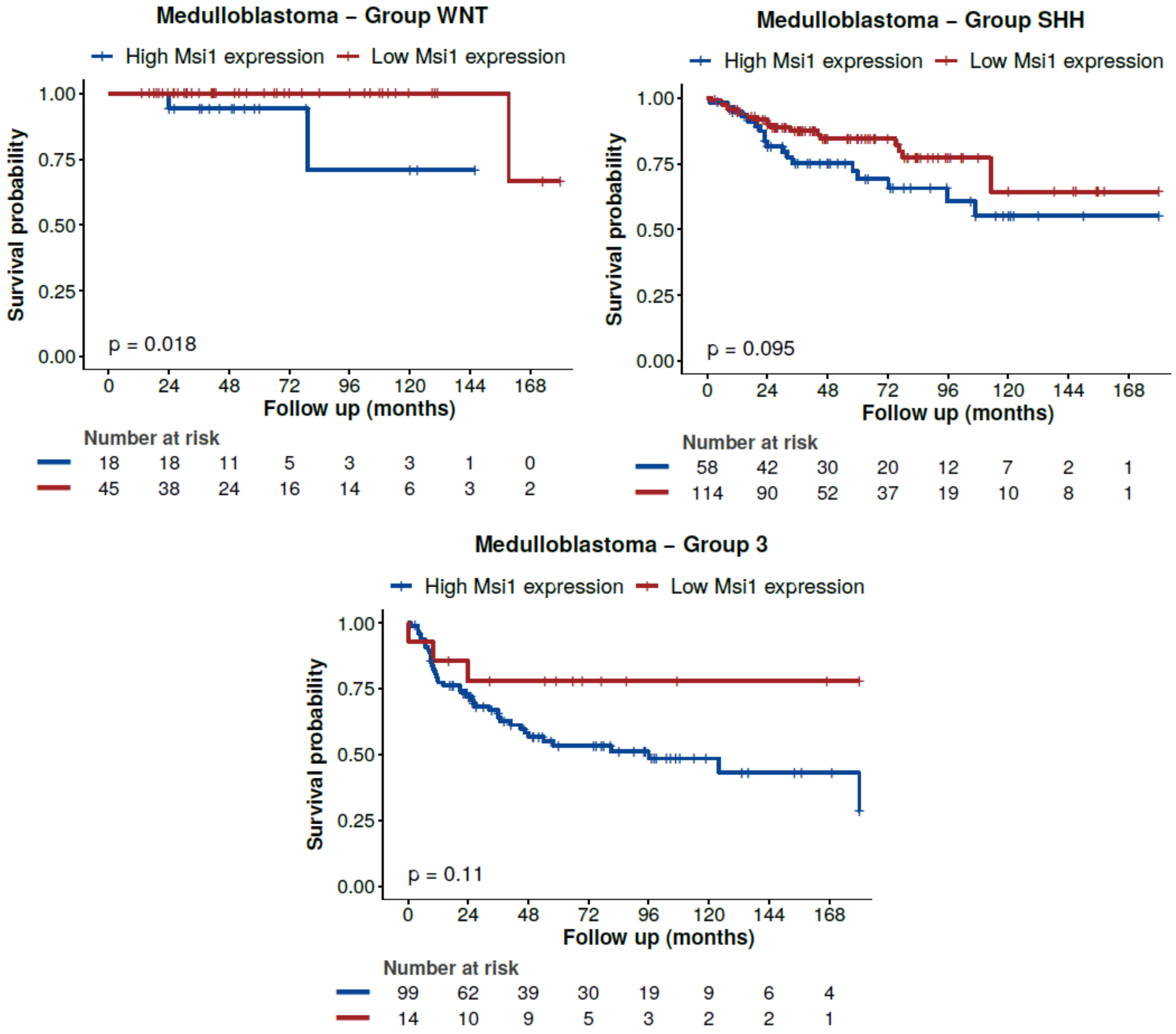

Figure S1. Kaplan-Meyer curves showing the impact of Msi1 expression levels on the survival of MB patients from Groups WNT, SHH and 3. The number of patients at risk on each time interval is indicated below Kaplan-Meyer curves.

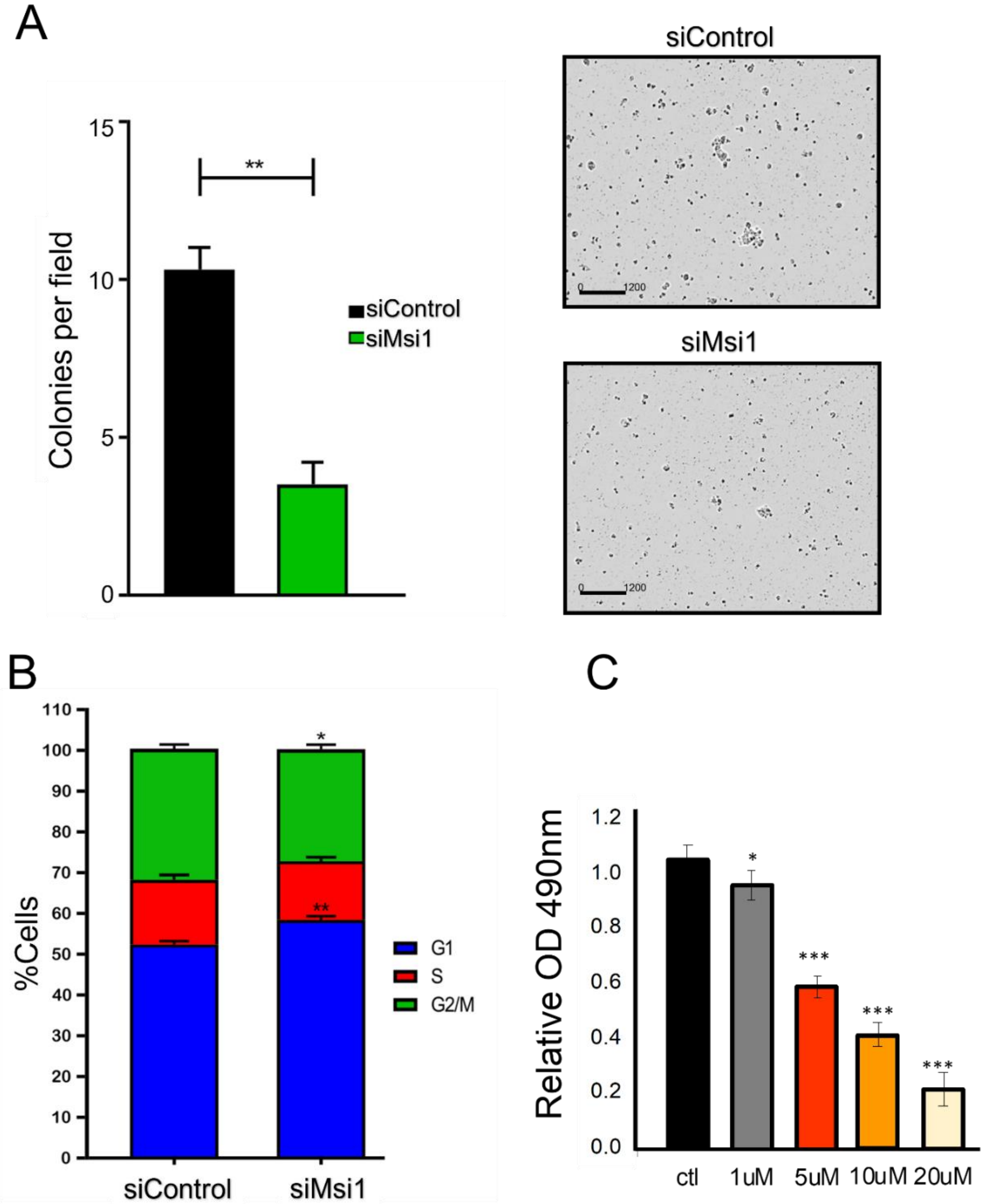

Figure S2. Msi1 impact on CHLA-01 cells phenotypes. Effect of Msi1 knockdown on cell growth (A) and cell cycle distribution shown in FACS sorted cells (B). C) Cell viability measured with the MTS assay comparing control and treated cells with Luteolin. Statistical significance calculated by one-way ANOVA and t test. Data shown as means ± s.d (*P<0.05, **P<0.01, ***P<0.001 **** P<0.0001).

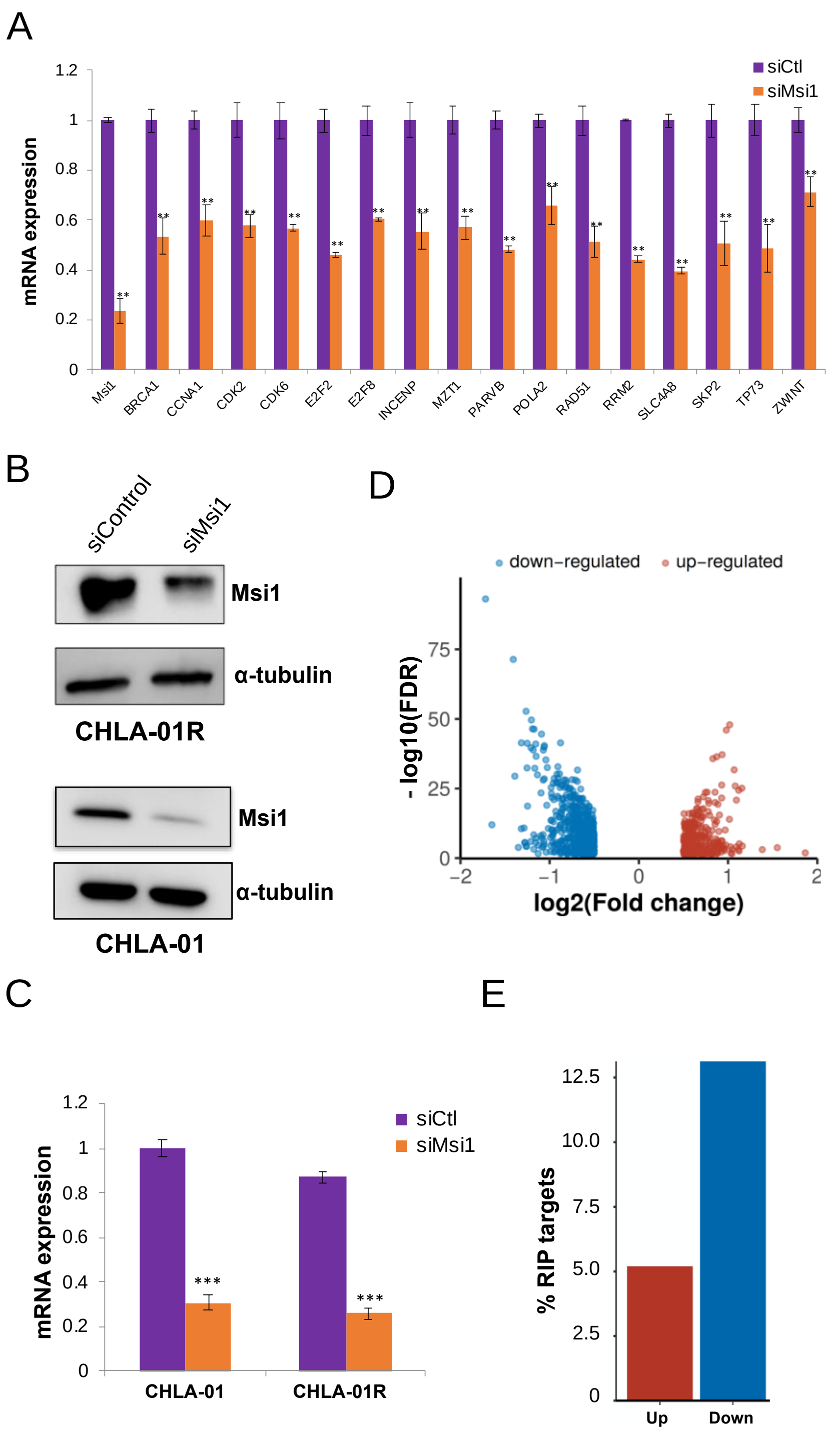

Figure S3. Confirmation and validation of Msi1 knockdown in CHLA-01 and CHLA-01R cells. A) qRT-PCR analysis of cell cycle/division genes using a different Msi1 siRNA in CHLA-01R cells. Analysis of Msi1 levels by western blot (B) and qRT-PCR (C) after Msi1 siRNA transfection in CHLA-01 and CHLA-01R cells. D) Volcano plot with up- and down-regulated genes from RNA-Seq analysis. E) Percentage of target genes identified by RIP-seq that also appeared in the down or up regulated sets.

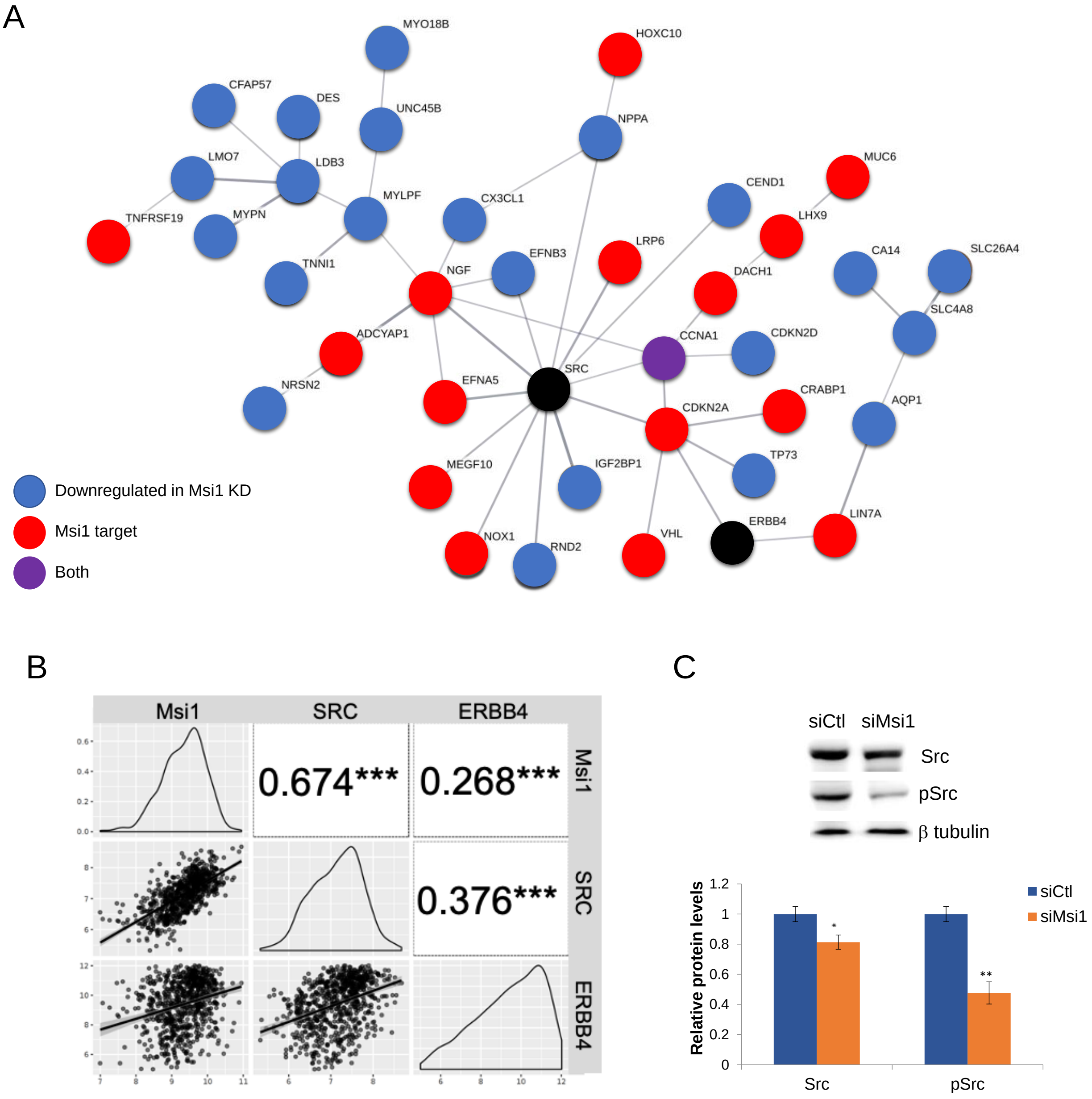

Figure S4. Musashi1 relationship with SRC and ERBB4. A) Network showing genes regulated by Msi1 associated with ERBB4 and SRC according to STRING [1]. B) Expression correlation analysis in the Cavalli medulloblastoma dataset [2] using Gliovis resources [3] C) Protein expression of Src and p-Src after Msi1 knockdown in CHLA-01R cells by Western blot. (* $p< 0.05$ ** $p<0.01$ *** $p<0.001$).

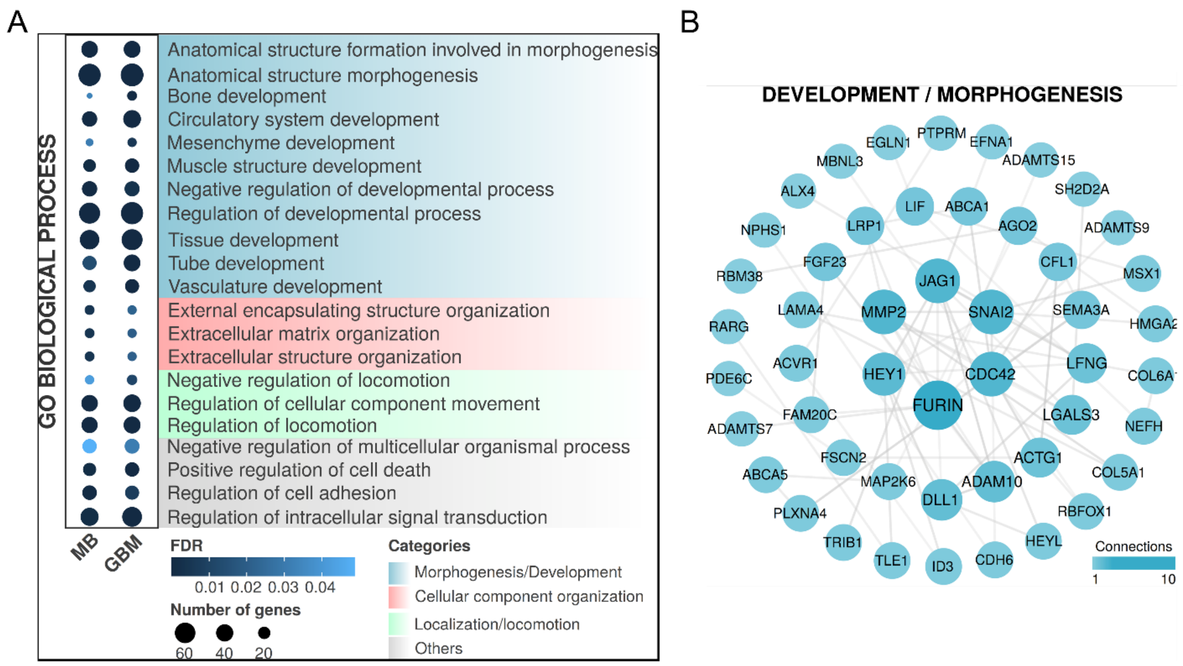

Figure S5. Musashi1 regulates a network of genes implicated in development/morphogenesis in medulloblastoma G4 cells. A) Common gene ontology enriched terms (biological processes) associated with genes upregulated after Msi1 knockdown in CHLA-01R cells and genes upregulated in GBM Msi1 KO lines [4]. B) Protein-protein network according to STRING [1] showing development/morphogenesis showing increased expression after Msi1 knockdown.

SUPPLEMENTARY TABLES

Table S1. List of primers and probes used in qRT-PCR analysis.

Table S2. Genes altered in CHLA-01R Msi1 knockdown cells identified by RNA-seq. Sheet 1) Summary; Sheet 2) Genes showing changes in expression after Msi1 knockdown; Sheet 3) Gene Ontology analysis of downregulated genes after Msi1 knockdown; Sheet 4) Gene Ontology analysis of upregulated genes after Msi1 knockdown; Sheet 5) Common GO terms (in red) identified in the comparison to enriched GO terms identified in the analysis of upregulated genes in Msi1 KO GBM cells [4].

Table S3. Musahi1 target genes in CHLA-01R cells identified by RIP-seq. Sheet 1) Results of the RIP-seq analysis; Sheet 2) Msi1 identified targets; Sheet 3) Gene Ontology analysis of coding transcripts associated with Msi1; Sheet 4) Comparison between Msi1 targets identified in this study and previous studies performed by our lab [5-7]; Sheet 5) Cell cycle and division genes identified in the RNA-seq and RIP-seq studies.

Table S4. Gene Modules identified with HumanBase [8]. Sheet 1) Gene modules and their associated GO terms identified in the analysis with downregulated genes from the RNA-seq study; Sheet 2) Gene modules and their associated GO terms identified in the analysis with Msi1 targets from the RIP-seq study; Sheet 3) GO terms present in gene modules identified in both the RNA-seq and RIP-Seq analyses.
Table S5. List of genes that were characterized as biomarkers of different subtypes of MB according to Hovestadt et al., 2019 [9].

REFERENCES

1. Szklarczyk, D., et al., *STRING v11: protein-protein association networks with increased coverage, supporting functional discovery in genome-wide experimental datasets.* Nucleic Acids Res, 2019. **47**(D1): p. D607-D613.
2. Cavalli, F.M.G., et al., *Intertumoral Heterogeneity within Medulloblastoma Subgroups.* Cancer Cell, 2017. **31**(6): p. 737-754.e6.
3. Bowman, R.L., et al., *GlioVis data portal for visualization and analysis of brain tumor expression datasets.* Neuro Oncol, 2017. **19**(1): p. 139-141.
4. Baroni, M., et al., *Musashi1 Contribution to Glioblastoma Development via Regulation of a Network of DNA Replication, Cell Cycle and Division Genes.* Cancers (Basel), 2021. **13**(7).
5. Uren, P.J., et al., *RNA-Binding Protein Musashi1 Is a Central Regulator of Adhesion Pathways in Glioblastoma.* Mol Cell Biol, 2015. **35**(17): p. 2965-78.
6. Vo, D.T., et al., *The RNA-binding protein Musashi1 affects medulloblastoma growth via a network of cancer-related genes and is an indicator of poor prognosis.* Am J Pathol, 2012. **181**(5): p. 1762-72.
7. de Sousa Abreu, R., et al., *Genomic analyses of musashi1 downstream targets show a strong association with cancer-related processes.* J Biol Chem, 2009. **284**(18): p. 12125-35.
8. Greene, C.S., et al., *Understanding multicellular function and disease with human tissue-specific networks.* Nat Genet, 2015. **47**(6): p. 569-76.
9. Hovestadt, V., et al., *Resolving medulloblastoma cellular architecture by single-cell genomics.* Nature, 2019. **572**(7767): p. 74-79.
